# Prevalence and risk factors associated with under-five years children diarrhea in Malawi: Application of survey logistic regression

**DOI:** 10.1016/j.heliyon.2024.e29335

**Published:** 2024-04-06

**Authors:** Mohammed Omar Musa Mohammed

**Affiliations:** College of Business Administration in Hawtat Bani Tamim, Prince Sattam Bin Abdulaziz University, Al-Kharj, Saudi Arabia

**Keywords:** Survey logistic regression, MICS, Complex survey, Under-five children, Prevalence, Risk factors

## Abstract

**Background:**

Diarrhea is a leading cause of illness and mortality among children under five, posing a significant public health challenge in Malawi. The current study assesses the prevalence and risk factors linked to diarrhea among under-five children in Malawi.

**Method:**

The researcher used the Malawi Multiple Indicator Cluster Survey (MICS) 2019-20 as the dataset for this study. Due to the complex sampling design, survey logistic regression was used to accomplish the study objectives. The sample size was 15569 children who were aged under five.

**Results:**

The study found that the prevalence of diarrhea was 24.9%, with a higher percentage observed among children aged 12–23 months (38.5%) compared to other age groups. Additionally, children from the southern region had a higher prevalence of diarrhea at 27% compared to those from the northern region at 19.3%. Children from the poorest households also had a higher prevalence of diarrhea at 28.9% compared to those from the richest households at 22.6%. Furthermore, children with fever had a higher prevalence of diarrhea at 33.3% compared to those who did not have fever at 19.4%.

**Conclusion:**

The current study concluded that the prevalence of diarrhea was higher among children aged 12–23 months. Subsequently, policymakers should apply policies to reduce this high prevalence among this age group of children. In addition, the government needs special consideration in diarrhea control for children from the southern region because of the high prevalence of the disease compared to the other regions in Malawi. My study can help policymakers understand the scope and nature of the problem, which can notify the development of policies and programs intended to decrease the prevalence of risk factors and enhance child health outcomes.

## 1 Introduction

Diarrhea is a condition described by loose, watery stools that happen more frequently than common. Among children under the age of five, diarrhea is typically acute. It can be affected by factors such as viral, bacterial, or parasitic infections, as well as malnutrition and inadequate sanitation [1]. It has been reported that, in 2019, diarrhea was responsible for approximately 1.5 million deaths among children under the age of five globally [2]. Worldwide, there are approximately 1.7 billion cases of childhood diarrhea disease annually [3].In Africa, diarrhea is a foremost source of mortality among children under five, with an estimated 314,000 deaths per year [1]. According to UNICEF, diarrhea is a top reason of death among children under the age of five in the third world, with a predicted 525,000 deaths per year. Additionally, it is estimated that children in the third world experience an average of three occurrences of diarrhea per year [4]. In developing countries, approximately 1.8 million individuals die each year because of diarrhea and more than 80% of them are children aged below five [5,6].

The prevalence of diarrhea among children under five was found to be 27.7%, with a higher prevalence observed in rural areas compared to urban areas [7]. Another study reported a prevalence of 23% among children under five, with risk factors such as type of floor and sharing a restroom with neighbors being significantly correlated with diarrhea [8]. Additionally, a prevalence of 20% was reported among children under five, with factors such as sex and age of the child, weight of the child at birth, region, mother's age, and occupation status being affected by the diarrhea status [9]. In Malaysia, a study found a prevalence of 4.4% among under-five children, with factors such as ethnicity and usage of untreated water being significantly associated with diarrhea [10]. Meanwhile, another study reported a prevalence of 15.5%, with factors such as place of birth and mother's education being significantly associated with diarrhea [11]. A prevalence of 17% was reported in yet another study, with children aged less than two years having a higher prevalence of diarrhea compared to those less than six months [12]. Children residing in rural areas had 22% lower odds of diarrhea than their urban peers [13]. The prevalence of childhood diarrhea in sub-Saharan Africa was found to be high, with factors such as mother's age, wealth index, mother's education, mother's job, age of the child, time of initiation of breastfeeding, and time to get water source being significantly correlated with diarrhea [14]. Previous studies have also identified various risk factors for diarrhea among under-five, including the mother's education, lower socio-economic status, breastfeeding, place of residence, family size, number of under-five children in the family, mother's age, and mother's employment status [[15], [16], [17], [18], [19], [20], [21], [22]].

Vaccines have a significant impact on the prevention of diarrheal disease in children under five. The uptake of vaccines, particularly those targeting diseases such as rotavirus, can greatly reduce the incidence and severity of diarrheal episodes at the ages of under-five. Vaccines play a crucial role in building immunity against specific pathogens that cause diarrheal infections, thereby reducing the risk of infection and its associated complications. Additionally, promoting the uptake of vaccines through vaccination campaigns and routine immunization programs can contribute to the overall reduction of diarrheal disease burden in children under five [23].

Studying the prevalence and risk factors of under-five diarrhea is critical because it can help identify the underlying causes of the condition and develop effective prevention and treatment strategies. Accordingly, this study aims to assess the prevalence and identify the risk factors associated with diarrhea among children under five years old in Malawi.

## 2 Materials and method

### 2.1 Source of data

The dataset for this study was extracted from Malawi (MICS) 2019–20. The National Statistical Office (NSO) in collaboration with UNICEF and other partners conducted the survey. It is a nationwide delegate survey designed to deliver data about children and women in Malawi. The two-stage stratified sample design was used to draw the sample, with enumeration areas (EAs) selected in the first stage and households chosen in the second stage. The survey collected data on a variety of indicators, incorporating child health and nutrition, education, child protection, water and sanitation, and HIV/AIDS. Since Malawi (MICS) 2019-20 is a complex survey in design, it was deemed that survey logistic regression would be a proper method for the analysis because it takes the complex design aspects such as sample weight, clustering, and stratification, as well as correcting Standard Errors and Confidence Intervals.

### 2.2 Study variables

The outcomes covariate for this study is the child who had diarrhea in the previous two weeks. A binary outcome of 1 indicated that the child had diarrhea in the previous two weeks, while 0 indicated otherwise. Furthermore, the predictors were a child sick with fever in the last 2 weeks, place of residence, region, gender, age (in months), Body Mass Index (BMI), mother's education, Ethnicity of household head, and wealth index. These variables were used by many scholars [[24], [25], [26], [27], [28]]. The study used external environmental predictors for under-five diarrhea which is deemed an important procedure hence these factors play a significant role in the occurrence and spread of diarrheal diseases among young children. By analyzing and understanding these external environmental predictors, public health officials and policymakers can implement targeted interventions to prevent and control diarrheal diseases in children under five. Overall, considering external environmental predictors is crucial for developing effective strategies to address under-five diarrhea and improve the health outcomes of young children. Additionally, addressing external environmental predictors for under-five diarrhea can have a broader impact on the community, leading to improvements in public health outcomes beyond just individual cases. Therefore, focusing on external environmental predictors is often prioritized in public health strategies aimed at preventing and controlling diarrheal diseases among young children.

### 2.3 Statistical model

Survey logistic regression is a statistical technique utilized to examine data from complex survey designs. It is a type of logistic regression that considers the sampling design of the survey, including stratification, clustering, and weighting [29,30]. Survey weights are applied to correct for unequal probabilities of selection and non-response biases. Clustering, which arises when data is collected from groups or clusters, is addressed by adjusting standard errors to reflect the intra-cluster correlation. Stratification involves dividing the population into subgroups, and analysis can be stratified, or stratification variables included in models.

Survey logistic regression and ordinary logistic regression are similar in many ways [31]. Rao and Scott [32] disclosed that data coming from complex survey design was not analyzed using ordinary logistic regression.

This approach incorporates weighting the estimating equation. As a result, the response variable is defined as $y_{hij}$, which equals 1 if the event occurred in $h^{th}$ individual within $i^{th}$ household within $j^{th}$ primary sample unit; otherwise, it is 0. The total number of observations, denoted as n, can be calculated as the sum of $n_{hji}$ for each combination of h,j, and i, $n=\sum_{h=1}^{H}\sum_{j=1}^{n_{h}^{\prime }}\sum_{i=1}^{n_{hj}^{\prime }}n_{hji}$, where h=1,…,H, representing the strata, within each stratum, $j=1,\ldots ,n_{h}$, representing primary sampling units (PSUs), each PSU is further divided into $i=1,\ldots ,n_{hj}$, representing secondary sampling units (SSUs), comprehending $n_{hji}$ elements. The sampling weight for the ${hji}^{th}$ unit in the dataset is indicated as $w_{hji}$. These weights are determined based on the sampling probability at each stage. Specifically, the design weights are obtained by multiplying the household design weights by the inverse of the household response rate within each stratum.

Let $\pi_{hji}$ = $P\left(Y_{hji}=1/X_{hji}\right)$, denotes the probability of an event in the sample, and $w_{hji}$ represents the sampling weight in each sampling unit for hji−th.

$$\text{Therefore},\text{the}\;\text{survey}\;\text{logistic}\;\text{is}\;\text{given}\;\text{by}\;logit\left(\pi_{hji}\right)=\log\left(\frac{\pi_{hji}}{1-\pi_{hji}}\right)=\overset{´}{{\;X}_{hji}\beta }\text{,}$$

where $Y_{hji}$ is the outcomes variable, $X_{hji}$ is the covariate matrix, and β is the coefficients of regression. Pseudo-maximum likelihood estimation (PML) was adopted to estimate the unknown parameters [[33], [34], [35]]. More details about survey logistic regression can be found in the literature [[36], [37], [38], [39]].

## 3 Results

This study considered 15569 children aged under five of whom 49.8% of them were male, whereas 50.2% were female. Of the overall sample, 24.9% of them had diarrhea in the past two weeks while 75.1 of them did not experience the disease in the same period. Table (1) shows that 39.4% of the children had a fever in the previous two weeks, whereas 60.6% of them did not have a fever in the same period. Regarding place of residence, most of the children 87.8% were from rural areas, and 12% were from urban areas. Table (1) also displayed that 46.6% of participants were from the south region, 33% from the central region, and 20.3% from the north region. Furthermore, Table (1) disclosed that 60.1% of the children were aged less than 35 months, whereas 39% of them were aged more than 36 months. Regarding mother education, the majority, 60, had primary education. It is observed that 31.1% of the household heads were from Chewa ethnicity, 16% from Lomwe ethnicity, 12% were from Yao ethnicity,11.2% were from Ngoni ethnicity, 8.4% were from other ethnicity and 9.7% were from Tumbuka ethnicity. Table (1) revealed that 23.5% of the participants were from the poorest households, 20.9% were from poor households, 19.8 were from the middle households, 18.9% were from rich households and 16.9% were from the richest households.

Table 2 presents the association between the diarrhea status of children under five in the previous two weeks and various related factors (see Table 1). The prevalence of diarrhea was found to be similar for males and females, both at 25%. children who had fever in the previous two weeks had a higher prevalence of diarrhea at 33.3% compared to those without a recent fever at 19.4%. The prevalence of diarrhea was marginally higher in rural areas (25.1%) compared to urban areas (23.4%). Among different regions, the southern region had the highest prevalence of diarrhea at 27%, while the northern region had the lowest at 19.3%. The highest prevalence of diarrhea was observed among children aged 12–23 months at 38.5%, while the lowest was among children aged 48–59 months at 16.2%. Children of Yao ethnicity had the highest prevalence of diarrhea at 27.3%, while children from Tumbuka ethnicity had the lowest at 19.8%. Additionally, the prevalence of diarrhea was highest among children from the poorest households at 28.9% and lowest among children from the richest households at 22.6%.

**Table 1:** Descriptive statistics of the participants.

Characteristic	N	%
Child had diarrhea in the last 2 weeks		
Yes	3876	24.9
No	11693	75.1
**The child ill with a fever in the previous 2 weeks**		
Yes	6138	39.4
No	9431	60.6
**Place of residence**		
Urban	1896	12.2
Rural	13673	87.8
**Region**		
North	3164	20.3
Central	5144	33
South	7261	46.6
**Gender**		
Male	7751	49.8
Female	7818	50.2
**Age (in months)**		
0–11	3109	20
12–23	3206	20.6
24–35	3189	20.5
36–47	3052	19.6
48–59	3004	19.3
**Mother education**		
Pre-primary or none	1580	10
Primary	10511	67.5
Lower Secondary	1715	11
upper Secondary	1536	9.9
Higher	206	1.3
Vocation Training	21	1
**Ethnicity of household head**		
Chewa	4837	31.1
Tumbuka	1512	9.7
Yao	1869	12
Lomwe	2494	16
Tonga	634	4.1
Sena	925	5.9
Nkhonde	234	1.5
Ngoni	1751	11.2
Other ethnicity	1314	8.4
**Wealth index**		
Poorest	3652	23.5
Poor	3254	20.9
Middle	3089	19.8
Rich	2938	18.9
Richest	2636	16.9

**Table 2:** Prevalence distribution of diarrhea among under-five children.

Characteristic	Diarrhea status		*P*- value
	Positive N(%)	Negative N(%)	
Child ill with fever in previous 2 weeks			
Yes	2042(33.3)	4096(66.7)	<0.0001
No	1834(19.4)	7597(80.6)	
**Place of residence**			
Urban	444(23.4)	1452(76.6)	0.112
Rural	3432(25.1)	10241(74.9)	
**Region**			
North	610(19.3)	2554(80.7)	<0.0001
Central	1306(25.4)	3838(74.6)	
South	1960(27)	5301(73)	
**Gender**			
Male	1950(25.2)	5801(74.8)	0.451
Female	1926(24.6)	5892(75,4)	
**Age (in months)**			
0–11	883(28.4)	2226(71.6)	<0.0001
12–23	1233(38.5)	1973(61.5)	
24–35	776(24.3)	2422(75.7)	
36–47	498(16.3)	2554(83.7)	
48–59	486(16.2)	2518(83.8)	
**Mother education**			
Pre-primary or none	359(22.7)	1221(77.3)	<0.0001
Primary	2742(26.1)	7769(73.9)	
Lower Secondary	417(24.3)	1298(75.7)	
upper Secondary	326(21.2)	1210(78.8)	
Higher	29(14.1)	177(85.9)	
Vocation Training	3(14.3)	18(85.7)	
**Ethnicity of household head**			
Chewa	1288(26.6)	3549(73.4)	<0.0001
Tumbuka	300(19.8)	1212(80.2)	
Yao	510(27.3)	1358(72.7)	
Lomwe	678(27.2)	1816(72.8)	
Tonga	117(18.5)	517(81.5)	
Sena	238(25.7)	687(74.3)	
Nkhonde	47(20.1)	187(79.9)	
Ngoni	418(23.9)	1333(76.1)	
Other ethnicity	280(21.3)	1034(78.7)	
**Wealth index**			
Poorest	1055(28.9)	2597(71.1)	<0.0001
Poor	811(24.9)	2443(75.1)	
Middle	729(23.6)	2360(76.4)	
Rich	678(23.1)	2260(76.9)	
Richest	603(22.6)	2033(77.1)	

In Table 3, the model fit statistics including the Akaike information criterion (AIC), the Schwarz criterion (SC), and the negative of twice the log-likelihood (-2Log L) were calculated to compare the model with only an intercept, and the model with both intercept and covariates. The results indicate that the AIC, SC, and -2Log L values for the model with intercept and covariates were smaller than those for the model with intercept only. This suggests that the model with intercept and covariates provides a better fit for the data.

**Table (3):** Model fit statistics.

Criterion	Intercept only	Intercept and covariates
AIC	17502.998	16407.690
SC	17510.644	16629.418
-2Log L	17500.998	16349.690

Table 4 provides information on the likelihood ratio test, efficient score test, and Wald test for testing the significance of the explanatory variable. The results of all these tests indicate high significance with p-values less than 0.0001. This suggests that at least one of the parameters is not equal to zero, indicating a significant relationship between the explanatory variable and the outcome.

**Table (4):** Testing global null hypothesis β = 0.

Test	F value	NUM DF	DEN DF	*P*- Value
Likelihood Ratio	37.28	21.1114	22273	<0.0001
Score	23.76	25	1031	<0.0001
Wald	26.14	25	1031	<0.0001

Table 5 summarizes the findings from the multivariate survey logistic regression analysis. Estimates, Standard Errors, unadjusted odds ratio, and p-value were calculated. The study found significant associations between under-five diarrhea and the following factors: having a fever in the previous two weeks, region, and age. However, no significant associations were found between under-five diarrhea and factors such as place of residence, gender, mother's education, ethnicity of household head, body mass index, and wealth index.

**Table (5):** Multivariate logistic regression results.

Parameter	Estimates	SE	OR	Pr > |t|
Intercept	−1.422	0.196	0.24	<0.0001
**Child ill with fever in previous 2 weeks(Yes = Ref)**				
No	−0.347	0.028	0.71	<0.0001
**Place of residence (Urban = Ref)**				
Rural	−0.065	0.057	0.94	0.261
**Region(South = Ref)**				
Central	0.065	0.062	1.07	0.2932
North	−0.261	0.081	0.77	0.0014
**Gender (Male = Ref)**				
Female	−0.002	0.032	1	0.9589
**Age (in months) (48–59 = Ref)**				
0–11	0.316	0.054	1.37	<0.0001
12–23	0.721	0.055	2.04	<0.0001
24–35	0.068	0.056	1.07	0.2251
36–47	−0.386	0.055	0.69	<0.0001
**Mother education (Vocation Training = Ref)**				
Pre-primary or none	0.135	0.193	1.14	0.4845
Primary	0.295	0.186	1.34	0.1128
Lower Secondary	0.282	0.196	1.33	0.1517
upper Secondary	0.077	0.201	1.08	0.7021
Higher	−0.247	0.313	0.78	0.4300
**Ethnicity of household head (Yao = Ref)**				
Chewa	0.037	0.083	1.04	0.6546
Tumbuka	0.136	0.101	1.15	0.1790
Lomwe	0.118	0.088	1.13	0.1791
Tonga	−0.128	0.175	0.88	0.4657
Sena	−0.089	0.118	0.91	0.4498
Nkhonde	−0.003	0.201	1	0.9867
Ngoni	−0.081	0.094	0.92	0.3420
Other ethnicity	−0.082	0.101	0.92	0.4186
**Wealth index (poor = Ref)**				
Poorest	0.104	0.052	1.11	0.0450
Middle	−0.028	0.057	0.97	0.6233
Rich	0.009	0.556	1.01	0.8718
Richest	−0.105	0.083	0.9	0.2065
**Body Mass Index**	0.000024	0.000025	1	0.3351

Children who had a fever in the previous two weeks had a higher risk of diarrhea compared to those who did not have a fever (OR = 0.71). The region was also found to be significantly associated with the risk of under-five diarrhea. Children from the central region were 1.07 times more likely to have diarrhea compared to children from the southern region. However, the place of residence did not show a significant association with under-five diarrhea.

Age groups were significantly associated with the risk of under-five diarrhea. Children aged 12–23 months were 2.04 times more likely to have diarrhea compared to children aged 48–59 months.

## 4 Discussion

Diarrhea is a common illness among children under the age of five, especially in third-world countries. It is a main public health concern in Malawi as it represents one of the main sources of disease and death among children below the age of five. Subsequently, the government of Malawi, in collaboration with its partners, has implemented several interventions to address this issue, including the promotion of proper hygiene and sanitation practices, the provision of safe drinking water, and the use of oral rehydration therapy. However, there is still much work to be done to reduce the incidence and impact of under-five diarrhea in Malawi.

Factors that increase the risk of diarrhea include poor sanitation, lack of access to clean water, malnutrition, and weak immune systems. Other risk factors include exposure to contaminated food or water, poor hygiene practices, and overcrowding. Preventive measures such as hand washing, proper sanitation, and access to clean water can help reduce the incidence of diarrhea in children. Additionally, timely and appropriate treatment with oral rehydration therapy can help reduce the severity and duration of diarrhea episodes. Considering this, the current study aimed to assess the prevalence and risk factors associated with children under five years of children's diarrhea in Malawi. The study used secondary data from Malawi (MICS) 2019–20. Survey logistic regression is perceived as a valuable tool for analyzing data with complex sampling designs. It allows for the consideration of survey weights, clustering, and stratification to provide more accurate estimates and inferences.

The impact of COVID-19 on the prevalence and risk factors of diarrhea among children under the age of five is multifaceted. The pandemic has disturbed healthcare services, availability of clean water, and nutrition, potentially leading to a rise in diarrhea cases among young children. Moreover, alterations in hygiene practices and restricted access to healthcare could also impact the factors that contribute to diarrhea in this demographic. Ongoing research is crucial for gaining a comprehensive understanding of these implications.

The current study found that the prevalence of diarrhea among the under-five children was 24.9%. This rate is higher than those generated by many previous studies [9,10,[40], [41], [42], [43]].

In the present study, it was also found that the region was significantly associated with under-five diarrhea. This result is in line with previous studies [9,44]. The high prevalence and risk factors for diarrhea among those under five in the southern region of Malawi can be attributed to a combination of factors such as limited access to clean water, poor sanitation, inadequate healthcare infrastructure, climatic differences between the regions, and socioeconomic disparities. These factors contribute to a higher burden of diarrheal diseases in this region.

The current study found an insignificant association between body mass index and under-five diarrheas in Malawi this finding agrees with a study conducted by Ref. [7]. It was further found in this study that the place of residence was not significantly associated with under-five diarrhea which contradicts the findings of [45]. The prevalence of diarrhea among children under five was slightly higher among children from urban areas with a percentage of 23.4% compared to rural areas with 25.1%. Such a finding is supported by Ref. [46]. Furthermore, my study did not find a significant association between the ethnicity of the household and diarrhea of under-five children. This finding was contradicted by the results of a study conducted in Malaysia [10].

The current study revealed that there is a significant association between age groups and under-five children's diarrhea. This result was supported by many studies [12,47,48].

The prevalence of diarrhea in children aged 12–23 months was notably higher at 38.5% compared to other age groups. This association can be attributed to factors such as developing immune systems, increased adventurousness, exploring new foods, being more active, possibly walking, and differences in exposure to pathogens. Consequently, this age group needs more attention to reduce this high prevalence of diarrhea. Furthermore, my study did not find a significant association between gender and under-five children's diarrhea, aligning with [47] where the prevalence was almost the same among males and females.

Another finding of this study showed an insignificant association between mother education and under-five children's diarrhea. This finding is supported by previous studies [7,14,20]. Furthermore, my study did not find a significant association between the wealth index of the household and under-five children's diarrhea. Such a finding contradicts a study conducted in Malawi [9]. Also, it was found that the prevalence was high among children from the poorest households with a percentage of 28.9%. This high prevalence among the poorest households needs to be reduced by the government of Malawi by enhancing the income. Additionally, the current study found a significant association between fever and under-five diarrhea which conforms to findings generated by previous studies e.g., Refs. [43,49]. This finding indicated that it is crucial to monitor and manage fever in children with diarrhea to prevent further complications.

The generalizability of the current study's findings is limited by some constraints, the acknowledgement of which is crucial. A more thorough analysis of diarrhea progression causes, and specific pathogens, along with stratified analysis and temporal spatial patterns, could enhance comprehensiveness. Connecting findings with public health policies and ensuring methodological transparency would strengthen the study's validity and guide targeted interventions. Moreover, incorporating these considerations can offer a more comprehensive understanding of childhood diarrhea, guiding preventive and treatment measures. Another limitation of studying the prevalence and risk factors of under-five diarrhea is the potential for misclassification of diarrhea cases, as definitions and reporting methods can vary across studies and settings. Additionally, confounding variables such as socioeconomic status or access to healthcare may not always be fully accounted for, which can impact the accuracy of risk factor associations. One limitation of the current study is the potential for recall bias when collecting data from caregivers about past episodes of diarrhea. Additionally, the cross-sectional nature of many studies may not capture the dynamic nature of risk factors and their impact on diarrhea prevalence over time. Furthermore, the generalizability of findings from a specific geographic area to other regions or populations may be limited. Furthermore, another limitation is that covariates such as vaccination and breastfeeding were not included in this study and more than 50% of participants did not respond. Given that this study relied exclusively on the external environmental predictors, it is recommended for future work to integrate both the external and internal environmental predictors such as genetic predisposition, immune system status, nutritional status, and overall health condition to account for a broader analysis and hence provide deeper insights on the issue.

## 5 Conclusion

Under-five children's diarrhea is a primary public health concern in Malawi. It is one of the top origins of disease and death in children below the age of five. The prevalence of under-five diarrhea was found high at 24.9%. The policymaker needs to apply relevant strategies to reduce this high prevalence. There are many ways to reduce the high rate by following preventive measures such as acquiring developed sanitation, adopting an effective approach to clean water, and implementing appropriate hygiene practices. Such measures can notably decrease the rate of diarrhea in children. Vaccines besides specific diarrheal remedies are additionally obtainable and can help avoid illness. My study can serve as a resource for policymakers who are seeking evidence-based information to support their decision-making processes related to child health and development.

## Ethical approval and consent to participants

Since this study used publicly available data from the MICS (Multiple Indicator Cluster Surveys) that is freely accessible on the UNICEF website, it is possible that ethical approval was not required.

## Consent for publication

Not applicable.

## Data availability

All the data and materials are available on this URL “https://mics.unicef.org/surveys”.

## CRediT authorship contribution statement

**Mohammed Omar Musa Mohammed:** Writing – review & editing, Writing – original draft, Visualization, Validation, Supervision, Software, Resources, Project administration, Methodology, Investigation, Funding acquisition, Formal analysis, Data curation, Conceptualization.

## Declaration of competing interest

The authors declare the following financial interests/personal relationships which may be considered as potential competing interests:Mohammed Omar Musa Mohammed reports financial support, administrative support, and article publishing charges were provided by 10.13039/100009392Prince Sattam bin Abdulaziz University. Mohammed Omar Musa Mohammed reports a relationship with Prince Sattam bin Abdulaziz University that includes: employment. Mohammed Omar Musa Mohammed has patent pending to 12344. NA If there are other authors, they declare that they have no known competing financial interests or personal relationships that could have appeared to influence the work reported in this paper.
